# Genetics of Tobacco Use

**DOI:** 10.1186/1617-9625-2-8

**Published:** 2004-06-15

**Authors:** Nancy Nairi Maserejian, Athanasios I Zavras

**Affiliations:** 1 Department of Epidemiology, Harvard School of Public Health, Boston, Massachusetts, USA; 2 Department of Oral Health Policy & Epidemiology, Harvard School of Dental Medicine, Boston, Massachusetts, USA; 3 Ingenix Epidemiology, Newton, Massachusetts, USA

## Abstract

The worldwide prevalence of tobacco use is widespread, resulting in nearly 4.5 million deaths every year. Nicotine is addictive and has psychopharmacological effects that maintain the use of tobacco products. Several studies have documented a strong hereditary component to tobacco use. The present article reviews results from twin and adoption studies and proceeds to present association studies of specific genes that may be involved in tobacco use. Cholinergic receptor nicotinic beta polypeptide 2, serotonin receptor and transporter genes, dopamine receptor and transporter genes, and the cytochrome P450A6 gene are reviewed. Linkage studies help to identify regions of the genome that may be worth further investigation. The paper concludes with a discussion of the limitations of genetic research and the future of genetic epidemiology in this domain.

## Epidemiology of Tobacco Use and Dependence

Tobacco use and dependence are byproducts of a large, complex web of social, environmental and genetic influences. Tobacco dependence essentially results from prolonged exposure to the pharmacologic effects of nicotine. Although smoking has been popular for more than a century, the addictive qualities of nicotine were not acknowledged in scientific literature until the 1980s. Consequently, despite the knowledge that tobacco use carries considerable health risks [1,2], more than 1.1 billion people (one-third of the global adult population) use tobacco regularly [3]. Current global estimates indicate that 48% of men and 12% of women smoke cigarettes [4]. Transnational consumption differences indicate that smoking is decreasing in most developed countries yet increasing in most developing nations. In the United States, 25% of the total population aged 12 or older (56.3 million people) smoke cigarettes, 5.4% (12.1 million people) smoke cigars, 3.2% (7.3 million people) use smokeless tobacco, and 1.0% (2.3 million people) smoke tobacco in pipes [5]. Among adult smokers, many wish to stop smoking, but the addictive qualities of nicotine create a complex barrier. In the United States, 70% of current smokers express a desire to quit, yet only 4.7% of those who attempted to quit in the past year were able to maintain abstinence from smoking for 3–12 months [2].

Withdrawal difficulties are evident even in adolescent smokers who try to quit [6]. Indeed, the age of smoking initiation is declining and tobacco use is rising among youth in many countries [7]. There are a number of complex and inter-relating factors that predispose young people to smoke, and these vary among individuals and among different populations. Low prices, easy access, parental smoking, family conflicts, peer pressure, and the positive image of smoking through advertisement are all contributing factors for tobacco use initiation in adolescence.

## Characteristics of Tobacco Users

Although historically, adult tobacco use was linked to affluence [8], today, continuing tobacco consumption is associated with societal disadvantage indicators, such as low income and low education [9], and individual factors, such as race, use of alcohol or other illicit drugs, depression, and personality type [10]. There are differences in specific smoking behaviors across races and genders. In the United States, Hispanics and Asians have lower smoking prevalences (19% and 14% respectively) than Caucasians or African-Americans (about 25%) [2]. Reports indicate that African-Americans start smoking regularly at a later age and smoke fewer cigarettes, but smoke those with higher nicotine content [11-13]. In terms of gender, differences may exist in nicotine sensitivity and nicotine requirements. For example, after smoking similar cigarettes, women have significantly lower nicotine levels than men [14]. Also, many studies show that although men are more likely to be smokers, women tend to have greater difficulty quitting [10].

Certain personality or behavioral traits, such as depression, neuroticism, attention deficit and hyperactive disorder (ADHD), and novelty seeking are associated with tobacco use [10,15]. Novelty-seeking is characterized by stimulation-seeking and sensitivity to reward or reinforcement, and is associated with nicotine dependence not only in adults [16], but also in adolescents [17] and early age of smoking initiation [18]. Children and teenagers with a diagnosis of ADHD engage in more health-threatening behaviors such as smoking, and alcohol and substance abuse. Though the etiology of ADHD is not known, recent studies suggest that maternal smoking during pregnancy is a factor and that there is a strong genetic link to the disorder [19].

In general, individuals who are tobacco-dependent exhibit greater psychiatric comorbidities that may stem from a common etiologic basis involving neuro-transmitter pathways and genetics. A prime example comes from the considerable and consistent evidence that smoking, alcohol, and depression are linked. Compared to never smokers, smokers have more than twice the lifetime prevalence of major depressive disorder [20]. Prevalence of smoking increases with levels of current symptoms of depression [21] and smoking cessation relapse is associated with increased symptoms of depression [20]. Furthermore, individuals with a combined history of alcoholism and major depression are at a high risk to use smoking as a means of mood enhancement [22]. A common neurological thread to these disorders is the involvement of serotonin and dopaminergic depletion and their associated rewards of pleasurable feelings [10,23,24], which are discussed in the following section. Thus, genes involved in the dopaminergic system have been studied in relation to alcoholism, depression and many other psychiatric disorders, with positive results [25]. Considering that tobacco dependence is linked to such individual characteristics that are shaped not only by environment, but also by genetics [10], it is not surprising that studies have found a strong hereditary component to tobacco use [26,27].

## Psychoactive Components of Tobacco

Nicotine's addictive characteristics are known to primarily result from its ability to bind to receptors in the brain, which then increase concentrations of neurotransmitters such as dopamine and serotonin in reward regions of the brain. Dopamine, affected by many stimulants and addictive drugs, is involved in the mesolimbic reward pathway, which produces pleasurable feelings. Recent human drug-reversal trials confirm that nicotine mediates reinforcement from smoking via dopamine, and that smoking behavior can be manipulated within the same subjects in opposite directions by alternately stimulating and blocking dopamine [28].

Dopamine is secreted partly by the release of serotonin (5-HT), which is a key neurotransmitter in the reward pathway and a logical candidate to study in relation to smoking behavior. When nicotine binds to its receptors, serotonin secretion from the brain increases [29,30]. The reward pathway proceeds as serotonin stimulates enkephalin in the hypothalamus, which then inhibits GABA at the substania nigra, finally releasing a specified amount of dopamine at the nucleus accumbens, termed the "reward site" [31]. Another link between nicotine and serotonin has been seen in the rat hippocampus, where smoking clearly reduces serotonin binding to serotonin receptors 1 (5-HT1) and 5-HT1A [32]. Thus, nicotine not only increases serotonin release from the brain, but also decreases its reuptake in the hippocampus. Together, these effects make available a greater amount of serotonin, which ultimately stimulates dopamine release. During withdrawal from nicotine, serotonin release is indeed reduced [30], and changes in mood seen in nicotine withdrawal may partly result from diminished serotonin transmission [33]. Accordingly, it has been hypothesized that people with a functional deficit in this dopamine pathway may be more prone to nicotine dependence [27].

To confirm the notion that smoking and nicotine affect the dopaminergic system in the human brain, neuroimaging techniques, such as positron emission tomography (PET), have been used to directly measure physiologic, pharmacokinetic and pharmacodymanic events in the same person following nicotine administration. These studies have shown that nicotine has rapid pharmacokinetics, changing cerebral blood flow and brain metabolism [34,35]. Compared to non-smokers, smokers exhibit significantly increased pre-synaptic dopamine transmission in dopaminergic brain regions [36]. Furthermore, smoking may create a vicious cycle of reward and deficiency; a recent study found that smoking seems to inhibit dopamine receptor D3 mRNA expression, thereby diminishing the rewarding effect of dopamine, leading to a reward deficiency state and creating motivation for continued smoking [37]. Indeed, phenotypes of interest have been delineated with the help of PET studies. For example, in response to monetary rewards, striatal and dopaminergic activity is different for smokers and non-smokers. A possible explanation is that a smoker's brain interprets and reacts to rewards differently, and perhaps in a more impulsive and sensation-seeking manner, than a non-smoker's brain [38]. Another phenotypical finding is that changes in cerebral blood flow following nicotine administration are strongly correlated to self-reported craving for cigarettes and addiction as a motivation for smoking [34]. Thus, it seems apparent that nicotine's effects are linked to neuroanatomical systems involved in the dopaminergic pathway, as well as arousal and reward.

Complicating the epidemiology of tobacco dependence is the notion that nicotine may not be the only psychoactive component found in tobacco. Primary evidence for this notion comes from the observation that nicotine can evoke a behavioral response that lasts much longer than the presence of nicotine in plasma [39]. Fowler and colleagues (1996) observed that smoking, but not nicotine, decreases the brain levels of an important enzyme that breaks down dopamine, monoamine oxidase (MAO), by 30–40%, which may result in increases in dopamine levels [40]. Further PET studies indicate that the reduction in MAO B in smokers is most likely to occur gradually and require chronic tobacco smoke exposure [41].

Another reason to speculate that additional components of tobacco use are involved is the recent observation that cotinine, the primary metabolite of nicotine, is bioactive as well [42]. Compared to nicotine, cotinine has a long pharmacological half-life (15–19 vs. 2–3 hours) and may be 5–30 times less potent [39]. Recent studies observed that cotinine evokes nicotine-like pharmacological responses, such as antagonizing the effects of dopamine receptor agonists [39]. Considering that cotinine, like nicotine, can stimulate dopamine release, it is theoretically possible that cotinine could mediate the long-term behavioral actions of nicotine. However, many studies have ascribed no pharmacological action to the compound; even in levels as high as ten times that attained from cigarette smoking, it has been found to have no observable acute or withdrawal effects in humans [43]. Other possible compounds of tobacco may reinforce dependence by irritating the mouth, throat or bronchial tree, and thereby enhancing the addictive potential of nicotine through altered sensory cues [44]. Overall, while many components of tobacco continue to be investigated in their role in addiction, it is clear that nicotine is at the core of neuropharmacological actions for many users.

Essentially, smokers alter their tobacco use to maintain enough levels of nicotine to act on neurotransmitters; therefore, genetic variation in the psychological need for nicotine, the ability to metabolize it, and the pathway to its pleasurable effects, ultimately influences if and how people use tobacco. Indeed, as smokers pass through stages from initiation to dependence, there are many opportunities for genetic factors to influence behavior. Research suggests that certain genes may be unique to one stage of the smoking process, while others influence several smoking characteristics [45-47]. The following paragraphs present major positive findings in twin and adoption studies that predict the strong role of genetics. The review then proceeds to discuss the results of association studies for specific alleles involved in tobacco use, as well as genome-wide scans and linkage studies.

## Twin and Adoption Studies on the Genetics of Smoking Behaviors

The twin pair study, which has long been a popular research design to investigate the role of genetics in disease causation [48], provided early evidence that there is a hereditary component to tobacco use. In 1958, there was a report of increased frequency of smoking among pairs of monozygotic male twins, as compared to dizygotic twins [49]; this observation implicated genetics in the etiology of the habit. Since then, innovative research designs in twins have attempted to quantify the role of inheritance in various outcomes, including initiation, intensity (current amount smoked), persistence (years of smoking), dependence and inability to quit. Kendler and colleagues (1999) studied 1,898 female twins in an effort to identify risk factors for initiation and dependence. They found that initiation was associated with low education, religiosity, increased neuroticism, extroversion and mental health comorbidities. Nicotine dependence was associated with low education, extroversion, mastery, self-esteem, increased neuroticism, history of mood disorders and alcohol abuse [50]. A review of data from 14 different twin studies and more than 17,000 twins estimated that genetic factors account for 56% of the variance in smoking initiation and regular tobacco use, environmental (shared, familial) risk factors account for 24% and individual risk factors account for the remaining 20%. The authors note that genetic factors appear to be more prominent in the transition to nicotine dependence, where they account for approximately 70% of the variance, and shared environmental influences seem negligible [47]. Elsewhere, smoking persistence (the number of years of regular smoking) has been found to be substantially due to genetic factors, with similar effects for women and men of all ages [46], although the role of environment, and especially low education, is significant. To estimate the heritability of failed smoking cessation, Xian and colleagues (2003) studied 1,818 twin pairs of smokers that had experienced at least one unsuccessful attempt to quit; they found that genetics accounted for 54% of the variance in risk of failed cessation and 29.7% of the variance in the risk of nicotine withdrawal [51].

In general, twin studies of both adolescents and adults indicate that the role of genetics is greater in dependence and quantity smoked, than in initiation, where environmental influences prevail [45,52,53]. Initiation, like persistence, is largely influenced by important interactions between environmental and genetic elements [54]. The notion that both environmental and inheritance factors may co-exist, and the fact that this coexistence is difficult to disentangle, create the potential for confounding or bias in twin studies. To avoid this problem, Osler and colleagues (2001) conducted an adoption study using 840 families [55]. Their finding that smoking status in adult adoptees was significantly associated with the smoking status of the biological full siblings, who were reared separately, supports the results of twin studies and strengthens the notion that there is a genetic influence on smoking [56].

## Genetic Association Studies

The fact that smoking is a complex process involving many different molecular targets has led to proposals to study a variety of genes as modulators of smoking behavior. To identify exactly which gene variants are associated with smoking phenotypes, molecular epidemiology has primarily used the candidate gene approach. For complex psychiatric disorders such as substance abuse, no single genetic locus is itself the causal factor; rather, multiple alleles found at various loci interact to produce vulnerability to an outcome. Unfortunately, the ability to identify numerous alleles that each contribute small effects, or that have an effect only when other critical alleles are also present, is poor, and studies are often under-powered [57]. Still, variations in the genome that have functional consequences (e.g., an important protein changes in the amount, location, or structure of expression in carriers of that allele) can be studied with success given adequate sample sizes and plausible mechanisms of action [58].

Advances in technology, together with the unique ability of these genetic variations to facilitate gene identification, have resulted in a recent flood of detection of single nucleotide polymorphisms (SNPs), which are often implicated in genetic association studies. Another recent focus of genetic research is the study of haplotypes, which are specified variants in an entire region of a chromosome, rather than just at one locus or gene. A 'haplotype map' is currently being constructed, to better pinpoint areas of chromosomes that are of interest during future research. Further information and updates on genetic research can be found at the National Center for Biotechnology Information web site: .

To investigate variant alleles and their role in smoking behavior, studies have focused on polymorphisms involved with the pathway of pleasurable sensations conferred by tobacco use, such as the dopamine system, as well as those involved in the metabolization of nicotine, such as genes that code for proteins in the cytochrome P450 family. The prominent outcomes that have been studied are smoking initiation, progression to nicotine dependence, quantity smoked, and persistence of smoking. Increasingly, endophenotypes, such as initial sensitivity to nicotine, are also being considered to further the power and interpretation of studies.

## Neurotransmitter Pathways

Since the effects of nicotine on the dopamine pathway are relatively well understood, many studies have investigated genetic variations in the dopaminergic system. Genes for this neurotransmitter's synthesis, degradation, receptors, and transporters are logical candidates. At the same time, serotonin, norepinephrine, GABA (gamma amino butyric acid), opioid, and cannabinoid neurons all modify dopamine metabolism and dopamine neurons. For example, mu-opioid receptors are known to play an important role in mediating the effects of various substances, such as opioids [59,60], cannabinoids [61] and alcohol [62].

Recently, it was shown that mice lacking mu-opioid receptors do not obtain rewarding effects of nicotine and have reduced withdrawal symptoms, suggesting a functional interaction between nicotine, the opioid system, and dopaminergic mesolimbic activity [63]. It has been proposed that defects in various combinations of the genes for these neurotransmitters result in a Reward Deficiency Syndrome (RDS) and that such individuals are at risk for abuse of substances that provide unnatural rewards [64].

In addition to this dopamine pathway, other neuronal systems, including brainstem cholinergic, GABAergic, noradrenergic and serotonergic nuclei, are also affected by nicotine and may be involved in nicotine dependence [65]. Much of the current knowledge of these systems comes from observations of the behavior of mice with mutations. Thus, the mechanisms of neurotransmitters, such as norephinephrine, or other pathways that are independent of the dopamine pathway, such as those following nicotinic reception in the brainstem, are yet to be determined [65]. While animal models are critical to the understanding of genetic factors in complex behavior, the following discussion concentrates on studies of smoking behavior in human populations and their associations with neurotransmitters with known polymorphisms.

## Nicotinic Receptors

Reward pathways are initiated when nicotine binds to nicotinic acetylcholine receptors (nAchRs), which then mediate the positive and negative effects that follow. Two classes of these receptors have been identified and many subunits are expressed in key regions of the brain and the mesolimbic dopamine system. One receptor gene, CHRNB2 (cholinergic receptor, nicotinic, beta polypeptide 2, located on chromosome 1q21) is a functional candidate gene for nicotine dependence, since it seems to be essential for a number of reinforcing effects of nicotine in mice [65,66]. However, a study of four SNPs in CHRNB2 found no association with the polymorphisms or their estimated haplotypes with smoking initiation or nicotine dependence, using carefully selected samples of non-smokers and regular smokers [66]. Polymorphisms in several other receptor subunits have since then been identified [67,68]. If new polymorphisms prove to have functional significance, then these genes may be candidates to explore in future research in smoking behavior [56].

## Serotonin

### Serotonin Synthesis Neurotransmitter, Tryptophan Hydroxylase Gene (TPH)

Tryptophan hydroxylase (TPH) catalyzes the rate-limiting step in serotonin biosynthesis. Although the known polymorphisms in the TPH gene are in non-coding regions and are not thought to have functional consequence [69], two polymorphisms, C218A and C779A, have been linked to personality traits [70] and have been studied in association with smoking (Table 1) [71,72]. These polymorphisms were not found to be associated with smoking status or progression to nicotine dependence. Significant associations, however, were seen in smoking initiation variables, such as age at onset of smoking [72] and ever having smoked more than an entire cigarette [71]. Considering the lack of functional significance of the polymorphisms and the possibility of false positive results, it is likely that either the TPH markers are in linkage disequilibrium for an unknown functional polymorphism, that the action of TPH on smoking is indirect (i.e., through a personality trait), or that TPH plays a minor role in the large web of causal factors for smoking initiation [71].

**Table 1:** Epidemiologic studies of tryptophan hydroxylase (TPH) gene, serotonin transporter gene and tobacco use in human populations.

**First Author, Year**	**Population, Sample size**	**Gene**	**Statistically Significant (p < 0.05) Results**
Lerman 2001	USA N = 451	TPH A/A, A/C, C/C	A/A genotype is associated with younger age at smoking initiation

Sullivan 2001	USA N = 780	TPH C218A and C779A	Allele frequency, genotype, and haplotype are associated with smoking initiation

Lerman 2000	USA N = 185 smokers	Serotonin Transporter 5-HTTLPR S/*^a ^vs. L/L	Interaction between genotype and neuroticism in nicotine intake, dependence and smoking motivations; neuroticism pre-dicted these behaviors among smokers with the S allele, but not among those with the L allele (no significant main effects of genotype)

Hu 2000	USA N = 759	Serotonin Transporter 5-HTTLPR S/* vs. L/L	Interaction between genotype and neuroticism in smoking status, initiation and cessation; neuroticism predicted these behaviors among smokers with the S allele, but not among those with the L allele (no significant main effects of geno-type)

Ishikawa 1999	Japan N = 496 men	Serotonin Transporter 5-HTTLPR L/* vs. S/S	L allele predicts increased smoking^*b *^and greater number of cigarettes/day

Lerman 1998	USA N = 498	Serotonin Transporter 5-HTTLPR S/S, S/L, L/L	No significant differences by genotype

a. A * denotes an allele other than the one noted

b. Increased smoking refers to comparisons between current, former and never or non-smokers (reference group)

### Serotonin Receptor Gene

Although there have been no reports of association studies between serotonin receptor gene polymorphisms and smoking, studies have provided grounds to explore the relationship [33]. For example, the effect of a polymorphism in the 5-HT2C receptor gene (Cys23Ser) on the reward dependence trait was significantly modified by the presence of a dopamine polymorphism (DRD4 VNTR) in individuals [73].

### Serotonin Transporter Gene

Unlike the receptor gene, serotonin transporter gene (SLC6A4) polymorphisms have been studied in association with smoking behavior (Table 1). The serotonin transporter gene, on chromosome 17q12, regulates the reuptake of serotonin from the synaptic junction. Gene transcription is modulated by a polymorphism (5-HTTLPR) that results in a long allele (l) or a short allele (s), which occur in 57% and 43% of Caucasians, respectively [74]. The s allele is associated with reduced transcription, resulting in a lower 5-HTT uptake and more available serotonin [75]. In addition, the s allele is associated with neuroticism, an anxiety-related personality trait [74], in samples from the United States, Japan and Israel [15].

The hypothesis that the increased available serotonin in individuals with the s allele would protect against habitual smoking or help in the smoking cessation process has been tested. No association between the 5-HTT polymorphism and smoking was seen in a study of Caucasians and African-Americans [76]. In contrast, a study of Japanese men found that the l allele was observed significantly more often in smokers than in nonsmokers or ex-smokers [77]. Although the subjects in the U.S. study were recruited through ads and may not have been representative of all smokers, the Japanese study differs from other studies by combining the l/l and l/s genotypes (since the l allele frequency tends to be lower in Japanese, at roughly 19%). Therefore, more studies are needed to confirm that there is a main effect of the serotonin transporter gene polymorphism on smoking behavior.

Meanwhile, an interaction between the transporter's 5-HTT polymorphism and neurotic personality has been found in two studies of smoking behavior [15,78]. For individuals with the s allele, neuroticism is linked to increased nicotine intake, dependence, smoking for stimulation, and smoking to reduce negative affect. In contrast, neuroticism is not an important impediment to smoking cessation for individuals with the l/l genotype, although they may exhibit the personality as well. Thus, rather than influence smoking initiation, these genotypes affect dependence, ability to quit, and motivations for smoking, such as smoking to self-medicate mood disturbances [15]. More work is needed to clarify the role of polymorphisms in serotonin genes, so that drugs used in tobacco cessation, such as the serotonin uptake inhibitor fluoxetine, can be administered to those people who would be most likely to benefit.

### Dopamine Transporter Gene (SLC6A3)

Released dopamine is taken up by the presynaptic neuron via the dopamine transporter (DAT), which is the primary mechanism for dopamine clearance from the synapse in the midbrain. The DAT protein is encoded by the locus SLC6A3 on chromosome 5p15.3. At SLC6A3, there is a polymorphism involving a variable number of tandem repeats (VNTR) in the 3'-untranslated region, with repeat numbers ranging from 3 to 11. The most common allele in population studies is the ten-repeat allele (A10, about 70% in European, Caucasian or African-Americans), followed by the nine-repeat allele (A9, about 25% in European, Caucasian or African-Americans). However, there is some regional variation; A10 is seen in more than 90% of Asian or South American populations, and certain African populations have high frequencies of the A7 allele. Although the VNTR polymorphism does not affect the actual protein product, it may affect mRNA localization, transcript stability, regulation of protein synthesis, and expression [79]. Indeed, the polymorphism seems to alter translation of the DAT protein and decrease its availability [80-82]. Under certain conditions, a decrease in transporter proteins decreases the clearance of synaptic dopamine. As a result, the baseline "set point" of synaptic dopamine is higher, thereby increasing sensitivity to drug-induced dopamine surges, or decreasing desire for substances, such as nicotine, that would increase synaptic dopamine [82].

### Dopamine Receptor Gene (DRD)

The synaptic levels of dopamine may also be determined by the density of dopamine receptors, which involves the dopamine receptor gene (DRD). Although there are many dopamine receptors (D1–D5), each exhibiting polymorphisms, the D2 dopamine receptor gene has been the focus of research regarding substance abuse. This is primarily due to the lack of evidence that the other polymorphisms have functional significance or are related to other complex psychiatric disorders [83]. The D2 receptor gene is located on chromosome 11q.23, and several polymorphisms have been identified, most notably in the TaqI A allele (A1 and A2) and the TaqI B allele (B1 and B2). Studies suggest that there is linkage disequilibrium between the less common A1 and B1 allele variants [84-86]. There is considerable variation in the frequency of these alleles in different racial and ethnic populations, even within European-American samples [87]. The A1 allele has been correlated with a reduced number of dopamine binding sites in the brain [84,88-90], and with increased dopamine transporter levels [91]. Heterosis, which refers to the situation where heterozygotes for an allele (e.g., A1/A2) are more likely to exhibit a phenotype than either homozygote (e.g. A1/A1 or A2/A2), was reported in the effect of the DRD2 gene on D2 receptor density [92].

Another DRD polymorphism that seems to have functional significance and has been studied in relation to smoking is the DRD4 VNTR polymorphism, on chromosome 11p15.5. There are three common variants of two (D4.2), four (D4.4), and seven repeats (D4.7) [93], which occur within coding regions. The longer, seven-repeat variant appears to blunt the intracellular response to dopamine in vitro, as compared with the 2 and 4 repeat variants [94]. D4 receptors are structurally and pharmacologically very similar to D2 receptors and are localized to the same limbic brain structures that underlie reward feelings [95]. It has been found that D4 receptors in the nucleus accumbens (the reward site) may modulate excitatory transmission [96] and the sensitization of these pathways [97].

### The Role of the SLC6A3 and DRD in Tobacco Use

The hypothesis that people with altered transport or a reduced D2 receptor density have a deficit in their reward system and feel an enhanced reward when exposed to dopaminergic agents, thereby making them more prone to nicotine addiction, has been supported by epidemiologic studies (Table 2) [27,85,86,98-105]. However, the findings regarding behavioral phenotypes associated with certain alleles are contradictory and indicate that the exact role of dopamine genes is complex. Although explanations for inconsistent findings are discussed in a subsequent section, it is worth noting here that heterosis in the effects of dopamine genes may play a role [89,106,107].

**Table 2:** Epidemiologic studies of dopamine receptor gene (DRD2), dopamine transporter gene (SLC6A3) and tobacco use in human populations.

**First Author, Year**	**Population, Sample size**	**Gene**	**Statistically Significant (p < 0.05) Results**
Erblich 2004	USA N = 108 smokers	SLC6A3-9/*^a ^vs. 10/10	SLC6A3-9/* increases number of cigarettes/day
		DRD2 A1/*^a ^vs. A2/A2	SLC6A3-9/* & DRD2 A1/* exhibit greater cigarette craving reactions
			Additive interaction of both gene variants

Vandenbergh 2002	USA N = 595	SLC6A3-10/10, 9/10, 9/9	SLC6A3-9/* predicts increased smoking^*b*^

Yoshida 2001	Japan N = 332	DRD2 A1/A1, A1/A2, A2/A2	A2 predicts increased smoking

Wu 2000	USA N = 140 lung cancer cases and 222 controls	DRD2 A1/A1, A1/A2, A2/A2	DRD2 predicts increased smoking, pack-years and number of cigarettes/day
		DRD2 B1/B1, B1/B2, B2/B2	A1 & case status interact to increase risk of family history of smoking-related cancer

Beirut 2000	USA N = 970 Alcoholism study subjects and their first-degree relatives	DRD2 A1/* vs. A2/A2	No significant differences by genotype

Lerman 1999	USA N = 522	DRD2 A1/* vs. A2/A2	SLC6A3-9/* predicts non-smoking, older age at initiation of smoking, and longer previous quit attempts
		SLC6A3-9/* vs. */*	DRD2-A1/* predicts smoking only through interaction with SLC6A3

Sabol 1999	USA N = 1,107	SLC6A3-9/* vs. */*	SLC6A3-9/* predicts smoking cessation & low novelty-seeking
		DRD2 A1/* vs. A2/A2	

Spitz 1998	USA N = 283	DRD2 A1/* vs. A2/A2	DRD2 A1/* or B1/* predict increased smoking, younger age at initiation & fewer attempts to quit
		DRD2 B1/* vs. B2/B2	

Comings 1996	USA N = 1026 (479 of which are from literature)	DRD2 A1/* vs. */*	DRD2 A1/* predicts smoking

Noble 1994	USA N = 354	DRD2 A1/* vs. A2/A2	DRD2 A1/* predicts increased smoking

Smith 1992	USA N = 232	DRD2 A1/* vs. A2/A2	DRD2 A1/* or B1/* predict increased substance use
		DRD2 B1/* vs. B2/B2	

a. A * denotes an allele other than the one noted

b. Increased smoking refers to comparisons between current, former and never or non-smokers (reference group)

With this in mind, the SLC6A3-9 allele has been found to be associated with an increased susceptibility to tobacco use in some studies [103,104], but a decreased susceptibility in others [100,108]. Earlier studies comparing those who have smoked <100 cigarettes in their lifetime (designated as non-smokers) to either former or current smokers found that carrying at least one copy of the 9 allele decreases risk of smoking by almost 50% and is associated with a four-fold higher likelihood of successful smoking cessation [103,104]. The notion that SLC6A3 is associated with smoking cessation was also supported by a recent case-control analysis using 250 current smokers [109].

Opposing findings, in which the 9 allele significantly increases the likelihood of smoking, may be partly due to the distinction made between never-smokers (never inhaled the smoke from even one cigarette) and non-smokers (smoked 1–100 cigarettes in lifetime) [100]. This distinction is likely to be important, particularly if risk-taking personality is a factor. The notion that the SLC6A3-9 allele may increase risk is supported by a recent investigation into the endophe-notype of self-reported craving; smokers with at least one copy of the 9 allele had significantly greater craving reactions following a laboratory-induced stress [108]. Interestingly, despite disagreement regarding the direction of the association of the SLC6A3-9 allele, studies agree that there seems to be a significant interaction between this gene and DRD2-A1 in increasing susceptibility to tobacco use [103,108]. An acceptable postulated mechanism is that individuals with certain SLC6A3 genotypes have lower endogenous synaptic dopamine, and therefore a greater need to use substances such as nicotine to stimulate dopamine transmission. This unfavorable effect of decreased dopamine may be especially pronounced in people with DRD2-A1 genotypes, i.e., those with deficient receptor density.

In general, the evidence for the DRD2 TaqI A1 allele is more consistent and points to an increased risk of tobacco use. Studies have found significant main effects with younger age at smoking initiation [86,105], fewer attempts to quit [86,104], shorter duration of quit attempts, current or past smoking [27,85,105] and craving reactions [108], but not nicotine addiction scales [86]. Recent investigations have found that DRD2 is associated with withdrawal symptoms, such as difficulty in concentrating and sleeping, and that DRD3 is associated with age of initiation, heaviness of smoking, and depression during withdrawal [99]. Meta-analyses based on three studies suggest that the odds ratio for likelihood of drug abuse is 2.4 for individuals possessing an DRD2-A1 allele and 3.3 for those having a DRD2-B1 allele (p < 0.001 in both cases) [110]. The notion that the A1 allele increases susceptibility to tobacco use is supported pharmacologically by randomized trials of bupropion hydrochloride, a weak dopamine reuptake inhibitor effective in decreasing withdrawal symptoms and craving. Studies have found that bupropion attenuates withdrawal-related craving, irritability and anxiety only among subjects with the DRD2-A2/A2 genotype [111,112]. A plausible biological hypothesis is that dopamine reuptake inhibition during nicotine withdrawal is less pronounced in DRD2-A1 carriers, since they have a limited ability to stimulate postsynaptic dopamine receptors in the brain's reward regions [90].

The association between DRD4 variants and smoking has been studied independently of the DRD2 and SLC6A3 polymorphisms, but results are similarly complicated (Table 3). Regarding the main effects of DRD4 in smoking initiation, dependence and persistence, analyses of Caucasians have found no association [23,113,114], but an African-American sample showed that presence of at least one long (L, 6–8 repeats) allele conferred an OR of 7.7 (95% CI 1.5, 39.9) for risk of smoking and a significantly shorter time to first cigarette in the morning [113]. In a combined sample, a significant interaction was seen between depression and DRD4, in which depressed smokers homozygous for the short alleles of DRD4 were more likely to smoke to reduce negative feelings and to smoke in response to stimulation [23]. The notion that smoking cues (e.g., seeing a lit cigarette) are important in the mechanism of D4 variants was further supported by a recent study, in which only individuals with long-repeats alleles showed significant reactions to smoking cues, in particular increased craving and arousal, with decreased positive affect [114]. The exact mechanism of affecting the reaction to smoking cues has been speculated, but requires verification by molecular studies [114].

**Table 3:** Epidemiologic studies of dopamine receptor gene (DRD4) and tobacco use in human populations.

**First Author, Year**	**Population, Sample size**	**Gene**	**Statistically Significant (p < 0.05) Results**
Luciano 2004	Australia N = 769 single twins	DRD4 48-base pair repeat polymorphism	No significant association or linkage tests

Hutchinson 2002	USA N = 68	DRD4 L/*^a ^vs. S/S	Interaction between genotype and response to smoking cues: DRD4 L/*, but not S/S, have increased craving, attention, arousal, and decreased positive affect during exposure to smoking cues
			No main effect of DRD4 on novelty seeking, nicotine dependence, smoking history or response to smoking cues.

Lerman 1998	USA N = 231 smokers	DRD4 L/* vs. S/S	Interaction between depression and genotype: depressed smokers with S/S are more likely to practice stimulation smoking and negative-affect reduction smoking; effect not seen in L/* genotypes
			No main effect of DRD4 on depression, stimulation smoking, negative-affect reduction smoking or nicotine dependence.

Shields 1998	USA N = 430	DRD4 L/* vs. S/S	In African-Americans: L/* predicts increased smoking, shorter time to first cigarette in the morning and earlier age at smoking initiation. No L/* genotype smokers were abstinent 2 months after smoking cessation counseling, vs. 35% of S/S genotype smokers
			No significant association with genotype and smoking in Caucasians.

a A * denotes an allele other than the one noted.

Of note, a recent study simultaneously analyzed linkage and association of the DRD4 genotype to smoking, alcohol and novelty-seeking behaviors in a sibling-pair design [115]. In the second wave of the study, DRD4 had a significant effect on smoking status, but the authors admit that the number of statistical tests performed and the inconsistency of the gene effect over time invalidate this finding. Thus, with improved methods, considerable power, repeated measures for some phenotypes, and strengths of having gender-, age-, and ethnicity-specific analyses, results showed no evidence that DRD4 variation has any affects on the outcomes. Although family studies and linkage analysis are discussed in other sections of this review, it can be noted here that a combination of linkage and association analysis, as was done by Luciano and colleagues, is likely to offer greater power and validity [115]. Consequently, the argument for a role for DRD4 in tobacco use is considerably weakened.

### Dopamine Metabolizers

Polymorphisms in genes that code for dopamine-metabolizing enzymes have also been linked to smoking behavior [116]. For example, the gene for monoamine oxidase A (MAO-A) has a point mutation (G→C) polymorphism causing an MAO-A deficiency. This polymorphism has been linked both to abnormal aggressiveness [117] and elevated brain levels of serotonin and dopamine [118]. A study in smokers found that individuals with the MAO-A 1460 TT/TO polymorphism smoke a greater number of cigarettes compared to those with CC/CT/CO (RR: 2.9, 95% CI: 0.6–5.1, p = 0.013) [116]. The C allele was less frequent in heavy smokers (>20 cig/day) compared to light smokers (<10 cig/day) (RR: 0.3, 95% CI: 0.1–0.7, p = 0.012). Another enzyme, dopamine B-hydroxylase (DBH), was also found to be associated with amount smoked per day. However, this finding reached statistical significance only in women, and the effect was small (those with the DBH 1368 GG genotype smoked 3.8 fewer cig/day). Heavy smokers were more likely to have the DBH 1368A allele, compared to light smokers (RR 2.3, 95% CI 1.1–5.0, P = 0.024).

Since cigarette smoking reduces the activity of MAO-A [40,119] and other enzymes, the relationship between these factors is complex and needs clarification. Future studies may also like to consider functional polymorphisms in MAO-B and catechol-O-methyltransferase (COMT), which also metabolize dopamine [33].

### Nicotine Metabolization Pathways: CYP2A6

Genetically-based individual variation in the metabolization of nicotine presents an additional pathway by which polymorphisms may influence smoking behavior. In humans, approximately 70–80% of nicotine is metabolized into cotinine [42], primarily by the enzyme cytochrome P450 2A6 (CYP2A6) [120,121]. This enzyme, responsible for the clearance of numerous chemicals, is predominantly expressed in the liver but also in respiratory organs [121]. Considerable interindividual variability in the activity of CYP2A6 has been seen in studies of human liver microsomes [122-125], as well as phenotyping studies using various ethnic populations [126-129]. Specifically, greater than 30-fold variation in nicotine-to-cotinine Vmax values [120] was revealed in a study of human liver microsomes. Although environmental compounds can affect its activity, either inducing the enzyme (e.g., phenobarbital) or decreasing its expression (e.g., nicotine) [130,131], the CYP2A6 gene also expresses genetic polymorphisms that may contribute to the variation in nicotine metabolism and therefore smoking behavior.

Numerous alleles have been found for the CYP2A6 gene locus on chromosome 19 (19q12 – 19q13.2). The wild-type, which is fully functional to metabolize nicotine, is the CYP2A6*1 allele. The primary null alleles are the *2, *4, *5 alleles. Since these null alleles code for inactivity, individuals homozygous for these alleles cannot metabolize nicotine through the CYP2A6 pathway. Other defective alleles (*6, *7, *9, *10, *11, *12) have reduced activity. Furthermore, a duplicate wild-type allele has been found, which confers increased activity. The frequencies of the defective alleles are low (1–3%) in Caucasian and European populations, but more common in Asian populations, where approximately 15% carry a null allele [132,133]. Although allele frequencies in African-Americans have not been reported, this group tends to have a generally decreased nicotine clearance, which may be due to decreased CYP2A6 activity [134]. Also, males tend to have faster rates of nicotine metabolism than do women [120,135,136].

The finding that smokers adjust the amount and intensity of their smoking to maintain peripheral and central nicotine levels [137,138] provides the basis for the proposed mechanism by which the defective alleles influence smoking behavior. The hypothesis is that those who carry a variant allele maintain greater levels of nicotine in their system for longer time periods after a given dose of tobacco; therefore, they need less external sources of nicotine. An additional consequence of a slower metabolism of nicotine is that bothersome effects of nicotine may be more pronounced. Thus, people with defective alleles may be more sensitive to tobacco products and less likely to become regular or dependent users [120].

Studies testing this hypothesis confirm that after oral administration of nicotine [139] or cigarette smoking [140], those with the defective alleles, compared to homozygotes for the functional allele, have significantly higher plasma levels of nicotine and lower levels of cotinine (Table 4). Furthermore, studies have found that individuals with defective alleles smoke fewer cigarettes per day and are less likely to become nicotine dependent [141,142]. Specifically, one study of 296 Caucasian men and women found that smokers who carry the null *2 or *4 alleles smoked fewer cigarettes per day both currently (13.5 + 2.3 vs. 19.5 + 0.7, p < 0.03) and at the time of heaviest smoking (19 vs. 29, p < 0.001), had lower breath carbon monoxide levels, and had lower cotinine levels than did individuals homozygous with the wild-type *1 fully functional allele. Interestingly, people carrying CYP2A61/1 plus duplication had higher carbon monoxide and cotinine levels, but reported smoking fewer cigarettes per day, than did people with either a single copy of the active or defective alleles. The authors hypothesize that those with the duplicate gene smoke more intensely, rather than more frequently, in order to maintain their nicotine levels [141]. A separate study confirmed findings that nicotine and cotinine levels significantly vary by genotype according to level of enzyme activity associated with the allele. For example, people with the 1/1 genotype had the lowest plasma levels of nicotine and highest levels of cotinine, while people carrying the defective *7 allele had intermediate levels, and those carrying the null *4 allele had the highest levels of nicotine and lowest levels of cotinine [139].

**Table 4:** Epidemiologic studies of cytochrome P450 2A6 (CYP2A6), nicotine metabolization and tobacco use in human populations.

**First Author, Year**	**Population, Sample size**	**Gene**^*a*^	**Statistically Significant (p < 0.05) Results**
Xu 2002	Canada N = 14 in kinetic substudy N = 478 in allele frequency study	Wild-type CYP2A6*1/*1 vs. *4/*4, *4/*10, *4/*7, *7/*7, *1/*8	Homozygous wild-type genotypes CYP2A6*1/1 have lower plasma levels of nicotine and higher levels of the CYP2A6-mediated nicotine metabolite cotinine, compared to those with the null *4/4 genotype (6 hours after oral administration of nicotine)
			*7/*7 genotypes or *7 in combination with gene deletion have intermediate levels of nicotine
			One individual containing both *7 and *8 (CYP2A6*4/*10) has sharply reduced metabolism of both nicotine and cotinine

Rao 2000	Canada N = 296 smokers	CYP2A6*1/*1 vs. *1/*4, *1/2, *2/2, or *1/*1 plus duplication	Smokers with reduced activity or null alleles (*2/2, *1/2 or *1/4) use fewer cigarettes/day compared to those with wild-type (*1/*1) genotype both currently (13.5 ± 2.3 vs. 19.5 ± 0.7) and at time of heaviest smoking (19 vs. 29), and have lower cotinine Levels

Kitagawa 1999	Japan N = 11 smokers in smoking challenge sub-study N = 252 in genotype study	CYP2A6*1/1 vs. homozygous deletion	Homozygous wild-type genotypes CYP2A6*1/1 have higher cotinine concentrations in urine than homozygously deleted genotypes (average concentration 3.87 ± 1.64 vs. 0.40 ± 0.15 ③ g/ml at 1.5 hours after smoking for 1 hr.)
			On average over the 24-hr period following smoking challenge, cotinine excretion in homozygously deleted genotypes was one-seventh compared to control group (p < 0.001)

Pianezza 1998	Canada N = 428	CYP2A6*1/1 vs. null alleles *2 or *3	Dependent smokers have lower frequency of null alleles, compared to the never-dependent control group (12.3% vs. 19.6%, p < 0.04, OR = 1.74, 95% CI 1.02–2.94)
			Smokers heterozygous for null alleles have fewer number of cigarettes/week (129 vs. 159, t-test p < 0.02), compared to smokers with two active alleles

a. We have used the nomenclature system for CYP2A6 alleles recommended by the Human Cytochrome P450 Allele Nomenclature Committee (available at )

Other cytochrome P450 enzymes that have been found to be involved in the metabolism of nicotine include CYP2B6 and CYP2D6; however, their contributions are minor [120,121]. In human liver microsomes, CYP2B6 was found to be involved in nicotine C-oxidation at high, but not low, substrate concentrations. CYP2D6, on the other hand, is unlikely to have a role in nicotine metabolism, according to recent findings [121,143].

In summary, studies have found polymorphisms in CYP2A6 to be associated with risk of tobacco-dependence, amount smoked, various smoking indices, and tobacco-related cancer [133,144]. The complexity of the role of cytochrome P450 is increased by the fact that environmental chemicals and drugs may induce its activity, therefore affecting the overall clearance of nicotine from the body [120,131]. Another concern is that these polymorphisms are quite rare in Caucasian populations, thereby limiting the ability to study the role of CYP2A6 in nicotine metabolism and smoking behavior. However, if variant alleles do indeed affect metabolism and smoking behavior, then the efficacy of nicotine-replacement therapy can be greatly increased by identifying and using inhibitors of CYP2A6 [145-147]. Therapeutic use of CYP2A6 inhibitors to mimic the decreased activity of variants may produce the same benefits of the null alleles and provide new approaches to prevention and treatment of tobacco use.

## Linkage Studies

While the goal of association studies is to identify specific alleles, linkage analysis aims to identify regions of the genome likely to be involved in susceptibility to tobacco use. In 1999, a complete genome scan revealed that there are many regions with numerous consecutive markers that yield small but positive results of linkage to a dichotomous outcome of nicotine dependence [148]. However, further investigation into regions of chromosomes 2, 4, 10, 16, 17 and 18 failed to find any significant linkage. Despite inconclusive findings, the authors of this study re-analyzed this affected sibling pair data in 2004 for a hypothesis-generating study of nicotine dependence candidate genes, via linkage, epistasis and bioinformatics. The recent analysis suggests that the results of the genome scan were more informative than initially perceived, and indicates a possible etiological relevance for 10 genomic regions. In particular, a broad region on chromosome 2 (2q22.1–2q24.1) and a narrower region on chromosome 10 (10q23.33–10q25.1) nearly reached the rigorous Lander and Krugylak [149] criteria for "significant" linkage. The analysis also searched for genome-wide epistatic interactions and found four correlations of interest, one of which (correlation of D8S1145 with D22S444) was particularly large and unlikely to be due to chance. Interpretation of the epistatic correlations is hampered, however, by the fact that markers in each correlated pair did not have sizeable positive NPL Z scores [150].

To clarify the significance of these results and to form hypotheses on candidate genes in nicotine dependence, the authors looked for agreement between genes located in the 10 regions statistically implicated in their analyses with a list of genes suggested by microarray studies and human association studies. Correspondence was found for genes involved with the mitogen-activated protein kinase (MAPK) signaling system, nuclear factor kappa B (NFKB) complex, neuropeptide Y (NPY) neurotransmission, a nicotine receptor subunit (CHRNA2), the vesicular monoamine transporter (SLC18A2), genes in pathways linked to human anxiety (HTR7, TDO2, and the endozepine-related protein precursor DKFZP434A2417), and the mu-opioid receptor (OPRM1) [150].

Unfortunately, the results of this genome scan are not entirely in agreement with studies using data from the Collaborative Study on the Genetics of Alcoholism (COGA), which also found some evidence for linkage with tobacco use. In COGA, using a dichotomous outcome of ever/never smoker, a sib-pair analysis found support for linkage, particularly to regions on chromosomes 6, 9 and 14 [151]. The evidence for linkage was weaker when the analysis used a cumulative pack-year history as the outcome, although some candidate regions were still noted. Agreement between the findings of COGA and the genome scan of Sullivan and colleagues occurred in areas found to have only weak or equivocal linkage signal in the latter study, on chromosomes 4, 6, 9, 14 and 17. However, the COGA analysis does correlate to other study findings; two of the three markers shared between COGA's ever/never outcome and the pack-years outcome have been previously identified as associated with smoking; one is the locus for DBH, a smoking candidate gene, and the other is for the chromosome 5q marker D5S1354. The chromosome 5 marker was found to be the primary genetic determinant of smoking behavior in a separate COGA study published that year [152]. Interestingly, this locus is very close to the D1 dopamine receptor gene locus, which has been previously been associated with smoking [153]. Although these findings are intriguing, additional linkage and experimental studies need to clarify the relevance of these overlaps.

Linkage studies have potential to support the candidacy of the genes discussed in this review. Unfortunately, the analyses published thus far have not appreciably strengthened the evidence for these particular genes, such as DRD2 or CYP2A6. Disappointing results can be partly explained by the fact linkage studies have low power when testing genes with modest or small effects. A gene with a relative risk of 2 or less requires sample sizes that are rarely attainable (>2,500 families) to obtain 80% power [154]. Inconsistencies may also be due to the fact that the currently available genetic maps are still imprecise, despite the efforts of the Human Genome Initiative toward high-resolution physical maps and large-scale sequencing. As the number of known genes increases with time, directed genomic screening may at times become more appropriate than random genomic screening, which has been the primary method of linkage studies of tobacco use [155]. In the meantime, direct tests of association, which remain powerful despite modest relative risks, are useful tools [154]. To direct genomic screening towards the best candidates, association studies will need to find solutions to their own current limitations, as discussed in the following section.

## Limitations and Trends for Future Research in the Genetics of Tobacco Use

Overall, the role of genetics in determining tobacco use seems to be an important yet complex one. Much of the difficulty in identifying key genes is the fact that they are likely to be numerous, each with small effects that together shape various phenotypes associated with smoking. Despite small effects, genetic polymorphisms are of public health significance; their attributable risk is likely to be substantial due to their frequency in the population [154]. Unfortunately, studies of single genes will often suffer from insufficient power to find such small effects. Furthermore, there are limitations of past studies that make attempts to unify their results difficult, and these issues imply certain considerations that are necessary in future studies.

A primary concern here is that the exposure variable used in these studies, namely, one particular gene, may be insufficient by itself to detect true associations. That is, it is necessary to examine gene-gene interactions and gene-environment interactions. For example, while serotonin transporter or DRD4 receptors genes do not seem to have main effects on smoking, investigations considering their interaction with environmental factors found that these gene variants are important with certain personality types or when subjects are presented with smoking cues. In future studies, the insufficient power offered by studying one single locus may be partly overcome by studying haplotypes or the association of multiple polymorphisms within and around an unknown polymorphism of significance. While family studies are needed to construct haplotypes, association studies can use statistical methods to estimate haplotype frequencies. Either a single haplotype, or a subgroup of more than one haplotype, may be of greater frequency in cases than in controls, indicating haplo-type or allelic heterogeneity [58]. Implicated genes may then be investigated in animal models to establishing possible mechanisms of action.

A separate reason for reduced power in past studies may involve the recent finding that heterosis occurs with certain genes and smoking behavior [106]. This finding suggests that in future studies, comparing the frequency of heterozygotes and genotype distribution may have better discriminative power than comparing gene frequency [156]. Furthermore, false negatives may be reduced if gender differences and gender-specific heterosis are considered in the analysis of association studies [106]. In the D2 gene, for example, studies have found that the effects of variants are in opposite directions for males and females [89,106,107], and heterosis has been suggested by one study, where male heterozygotes had significantly higher smoking rates than male homozygotes, while female heterozygotes were less likely to smoke than female homozygotes [106].

Inconsistencies across studies are also likely to be due to false positives, as the risk of a type I error is alarmingly high when the candidate gene approach is used. The standard p-value of 0.05 may not be appropriate, and its use has led to controversy over how to view varying results of studies of complex behaviors [157]. The mistake of relying on statistical significance to determine the truth of a hypothesis is exacerbated when one considers other sources of bias that may also lead to false positives. Confounding issues need to be anticipated and properly controlled. Although population stratification was once thought to be an important concern in confounding, it is doubtful that it is the cause of much bias in properly designed genetic association studies [158], and if needed, it can be dealt with in the design phase by matching on race and ethnicity, or in the analysis by testing for its presence, followed by adjustment with genomic control (i.e., inflating the standard chi-square) or cluster analysis methods [159]. Indeed, failure to replicate findings of positive results is most likely to be due to issues in poor study design and execution, and investigator-reporting bias. Most notably, ascertainment and selection of cases and control subjects, sample size estimation, response rates, and laboratory and other measurement error need to be of prime concern for researchers [159].

A particular concern in the studies reviewed here is the choice of outcome variable. Many analyses use a crude dichotomy of smokers vs. non-smokers, paying no regard to light or heavy smoking and using various definitions of non-smokers. Also, it may be important to separate never users of tobacco (i.e., never smoked even one cigarette) from non-users (i.e., smoked more than one but less than 100 cigarettes) [80,100]. Indeed, some genes (e.g. CYP) rely on exposure to tobacco to determine the level of their expression or if they will even be expressed at all [160], making it is impossible to determine the effects of tobacco use when one has never been exposed. Another limitation of the exposure assessment in these studies is that only cigarette smoking has been taken into account; no information on smokeless tobacco, cigar, or pipe use has been incorporated into tobacco use variables. Misclassification of the outcome can obscure meaningful differences between groups.

Meanwhile, studies that have used more specific smoking outcomes are difficult to compare due to the wide variety of definitions used. Examples of phenotypes used as outcomes include age of initiation, time to nicotine dependence, quantity of use, frequency of use, or ability to quit. To facilitate interpretation of findings and increase statistical power to detect associations, there has recently been a movement towards the use of endophenotypes as outcomes in genetic studies. An ideal smoking endophenotype would be one that is narrowly defined, readily identifiable, empirically related to the clinical manifestation of nicotine dependence, and associated with an underlying biological mechanism [161]. Examples include initial sensitivity to nicotine, abstinence-induced withdrawal and response to different drug challenges [56].

In conclusion, to refine the accepted role of genetics in tobacco behavior, there is a need for studies that examine multiple genetic polymorphisms and that obtain various, detailed smoking-related phenotype information. Problems of misclassification, low power, and false positives are important not only in and of themselves, but also since they may contribute to the separate problem of publication bias, in which there is a tendency to publish only positive findings. Still, positive findings from family studies were able to stimulate association studies of genes and smoking behavior. Thus far, results in the serotonin transporter gene, dopamine transporter gene, dopamine receptor genes D2 and D4, and cytochrome P450 2A6 have provided the most compelling evidence of a connection with smoking behavior. Future association studies, coupled with linkage analysis in family studies, will provide deeper insight into the relationship between genes, the environment and tobacco use. Undoubtedly, knowledge of the underlying biological processes involved in the addiction of various smokers will lead to the identification of different types of smokers. Patient-specific therapy and therefore more effective and efficient treatment for tobacco dependence will soon follow.

## Competing interests

The authors declare that they have no competing interests.
